# An Ocean Acidification Acclimatised Green Tide Alga Is Robust to Changes of Seawater Carbon Chemistry but Vulnerable to Light Stress

**DOI:** 10.1371/journal.pone.0169040

**Published:** 2016-12-29

**Authors:** Guang Gao, Yameng Liu, Xinshu Li, Zhihua Feng, Juntian Xu

**Affiliations:** Marine Resources Development Institute of Jiangsu, Huaihai Institute of Technology, Lianyungang, China; University of Connecticut, UNITED STATES

## Abstract

*Ulva* is the dominant genus in the green tide events and is considered to have efficient CO_2_ concentrating mechanisms (CCMs). However, little is understood regarding the impacts of ocean acidification on the CCMs of *Ulva* and the consequences of thalli’s acclimation to ocean acidification in terms of responding to environmental factors. Here, we grew a cosmopolitan green alga, *Ulva linza* at ambient (LC) and elevated (HC) CO_2_ levels and investigated the alteration of CCMs in *U*. *linza* grown at HC and its responses to the changed seawater carbon chemistry and light intensity. The inhibitors experiment for photosynthetic inorganic carbon utilization demonstrated that acidic compartments, extracellular carbonic anhydrase (CA) and intracellular CA worked together in the thalli grown at LC and the acquisition of exogenous carbon source in the thalli could be attributed to the collaboration of acidic compartments and extracellular CA. Contrastingly, when *U*. *linza* was grown at HC, extracellular CA was completely inhibited, acidic compartments and intracellular CA were also down-regulated to different extents and thus the acquisition of exogenous carbon source solely relied on acidic compartments. The down-regulated CCMs in *U*. *linza* did not affect its responses to changes of seawater carbon chemistry but led to a decrease of net photosynthetic rate when thalli were exposed to increased light intensity. This decrease could be attributed to photodamage caused by the combination of the saved energy due to the down-regulated CCMs and high light intensity. Our findings suggest future ocean acidification might impose depressing effects on green tide events when combined with increased light exposure.

## Introduction

Due largely to burning fossil fuel and change of net land use, the carbon dioxide level in the air has increased by 40% since 1750 [1]. Meanwhile, the ocean—as a sink of carbon—has absorbed about 30% of the emitted anthropogenic carbon dioxide, leading to ocean acidification (OA) [1]. The pH of surface seawater has decreased by 0.1 since the beginning of the industrial era, corresponding to an increase of 26% in hydrogen ion concentration. A global increase in OA for all representative concentration pathway (RCP) scenarios is projected by Earth System Models, in which the corresponding decrease in surface ocean pH by the end of this century is in the range of 0.06 to 0.07 for RCP2.6, 0.14 to 0.15 for RCP4.5, 0.20 to 0.21 for RCP6.0, and 0.30 to 0.32 for RCP8.5 [1]. Such an increase in OA would also affect the stability or the baseline of carbonate chemistry in coastal waters, where most of the macroalgae inhabit. Furthermore, coastal waters are more susceptible to acidification than the pelagic ocean due to eutrophication and following microbial respiration [2].

The concentration of CO_2_ in seawater is vital for algal photosynthesis since ribulose-1, 5-bisphosphate carboxylase/oxygenase (Rubisco) can also catalyse ribulose-1, 5-bisphosphate with oxygen (O_2_) in a process called photorespiration if the ratio of CO_2_ to O_2_ is low [3]. Although the concentration of dissolved inorganic carbon (DIC) is very high (about approximately 2 mmol L^-1^) in seawater, its predominant form is HCO_3_^-^, and CO_2_ accounts for less than 1% of it. Rubisco has a relatively low affinity for CO_2_ and is consequently less than half saturated under current CO_2_ levels for most algal species [4,5]. Accordingly, most algae including macroalgae have evolved CO_2_ concentrating mechanisms (CCMs), which can promote the internal CO_2_ concentrations to the levels significantly higher than extracellular concentrations. On the other hand, a few red and green macroalgae do not have any CCMs at the Rubisco active site and acquire Ci solely by CO_2_ diffusion from the external environment [5,6]. Therefore, the influence of ocean acidification and a changed seawater carbonate system on macroalgae may be species specific.

Species that can actively use bicarbonate seem unlikely to show stimulation of photosynthesis or growth with an increase in atmospheric CO_2_ levels since increased atmospheric CO_2_ will result in a small proportional change in HCO_3_^-^ compared to CO_2_ and CO_3_^2-^ concentrations in seawater [7]. On the other hand, species relying on CO_2_ uptake by passive diffusion might benefit more than those that can take up CO_2_ actively with an active CCM. For instance, increasing atmospheric CO_2_ concentrations have been demonstrated to enhance the growth of the red algae *Porphyra yezoensis* grown at 15°C and 300 μmol photons m^-2^ s^-1^ light level [8], *Gracilaria* sp. grown at 20°C and 300 μmol photons m^-2^ s^-1^ light level [9], *Lomentaria articulata* grown at 10°C and 40 μmol photons m^-2^ s^-1^ light level [6], *Hizikia fusiforme* grown at 20°C and 120 μmol photons m^-2^ s^-1^ light level [10], and *Neosiphonia harveyi* grown at both 10°C and 17.5°C with the light level of 100–150 μmol photons m^-2^ s^-1^ [11]. On the other hand, negative effects of increased CO_2_ on photosynthesis in *Ulva* spp. cultured at 25°C and 200 μmol photons m^-2^ s^-1^ light level [12], as well as growth in *Gracilaria tenuistipitata* cultured at 25°C and 200 μmol photons m^-2^ s^-1^ light level [13], *P*. *leucostica* cultured at 15°C and 60 μmol photons m^-2^ s^-1^ light level [14], *P*. *linearis* cultured at 15°C and 100 μmol photons m^-2^ s^-1^ light level [15] and *Fucus vesiculosus* cultured at 10°C and 70–100 μmol photons m^-2^ s^-1^ light level [16] were observed. In addition, a *p*CO_2_ level of 750 μatm had no significant effect on the growth of 14 macroalgae species from three major divisions, i.e. Chlorophyta, Rhodophyta and Phaeophyta [17]. Recent research demonstrated that *U*. *rigida* grown at 13°C and 50 μmol photons m^-2^ s^-1^ light level [18] and giant kelp *Macrocystis pyrifera* grown at 12°C and 110 μmol photons m^-2^ s^-1^ light level [19] were also insensitive to ocean acidification (~1220 μatm *p*CO_2_).

**Fig 1:**
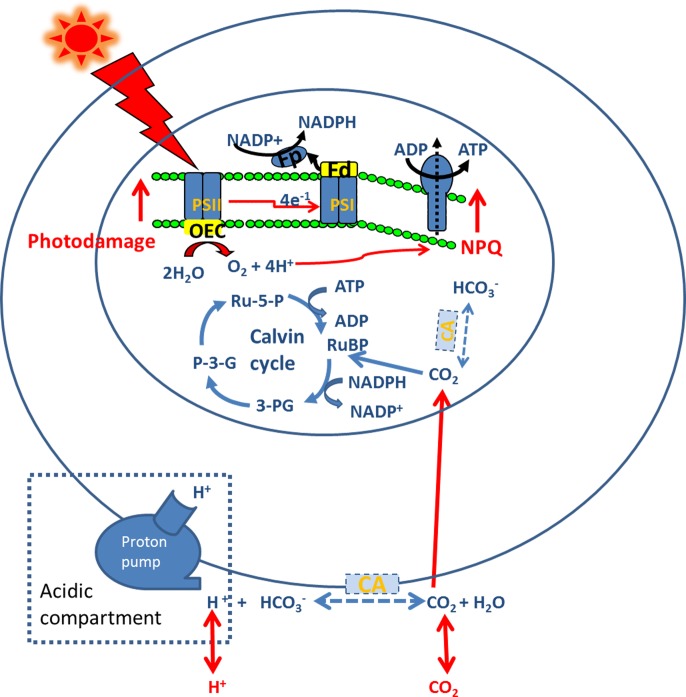
Physiological responses of ocean acidification acclimatised *U*. *linza* to changes of seawater carbon chemistry and light exposure. The blue-dotted and the red-solid symbols represent down- and up-regulated metabolic pathways respectively. PSI: Photosystem I; PSII: Photosystem II; Fd: ferredoxin; Fp: flavoprotein; OEC: oxygen- evolving complex; NPQ: non-photochemical quenching; CA: carbonic anhydrase.

*Ulva*, a cosmopolitan genus which is common in tide-pools, has been gaining significant interest due to its potential value in animal feed [20,21], human food [22,23], biofuel [24,25] as well as the delivery of crucial ecosystem services such as wastewater bioremediation [26,27] and CO_2_ removal [28,29]. Meanwhile, *Ulva* is the only genus contributing to reported green tide events [30,31]. A powerful CO_2_ concentrating mechanism (CCM) for algae is helpful to outcompete other species, particularly when CO_2_ is limited. *U*. *lactuca* has very efficient CCMs, including a HCO_3_^- ^dehydration mechanism and a HCO_3_^-^ uptake mechanism [32,33]. *U*. *prolifera* also has a high pH compensation point of 10.56, suggesting that thalli can utilise HCO_3_^-^ for photosynthesis [34]. However, little has been known about the CCMs in *U*. *linza*, a causative species of green tide [35,36], or the impacts of ocean acidification on CCMs in *U*. *linza*. Ocean acidification caused by increasing *p*CO_2_ can usually down-regulate algal CCMs and the saved CCMs-related energy expenditure facilitates growth [37,38]. On the other hand, the saved energy from down-regulated CCMs could harm photosynthesis and growth of phytoplankton when cells were exposed to increased light intensity [37]. Based on the previous studies, we hypothesise that ocean acidification will down-regulate the CCMs in *U*. *linza* and this down-regulation will not affect its effective response to seawater carbon chemistry but increase its sensitivity to high light (Fig 1).

## Materials and Methods

### Materials and culture conditions

Thalli of *U*. *linza* were collected from the coastal water of Lianyungang (119.3°E, 34.5°N), Jiangsu province of China in April 2013. No specific permission is required for the collection as *Ulva* is a nuisance in China. The natural temperature and salinity in the seawater were 12°C and 31 respectively. The thalli were transported to the lab in a cooling box (4–6°C) within one hour and then washed gently with 0.2 μm filtered seawater to remove any sediment, epiphytes or small grazers. The thalli were cultured in 500 mL conical flasks, aerated with ambient (LC, 390 μatm) and elevated (HC, 1000 μatm) CO_2_ levels. The culture densities for LC and HC treatments were controlled below 0.1g L^-1^ in order to maintain the pH variation less than 0.05. Media were made from natural seawater with the addition of 60 μM NaNO_3_ and 8 μM KH_2_PO_4_. The concentrations of nitrate and phosphate in natural seawater were 20 and 2 μM respectively and the artificial addition of nitrate and phosphate was to avoid the limit of nutrients during the laboratory culture. The light conditions were set as 100 μmol photons m^-2^ s^-1^ (light/dark 12:12 h) and the temperature was set as 12°C, in accordance with where *U*. *linza* was found in its natural conditions. The increased biomass was removed and the culture medium was renewed every 48 h. To avoid the effect of cutting on thalli’s physiological performances, the removal was conducted by taking out some individual randomly rather than cutting off every individual in the flasks. The culture lasted three weeks and five replicates were conducted under each treatment. According to Eggert [39], the acclimation of seaweeds to environment can take place over the period of hours to days. Therefore, three weeks should be enough for thalli to acclimatise to the condition of ocean acidification.

### Carbonate chemistry parameters

The *p*CO_2_ in seawater was maintained by bubbling ambient air (390 μatm) and CO_2_ enriched air (1000 μatm), and the latter was achieved by a CO_2_ plant chamber (HP1000 G-D, Wuhan Ruihua Instrument & Equipment Ltd, China) with the variation of CO_2_ less than 5%. The seawater pH in flasks was recorded with a pH meter (pH 700, Eutech Instruments, Singapore) and total alkalinity (TA) was measured by titrations. Other carbonate system parameters, which were not directly measured, were calculated via CO2SYS [40], using the equilibrium constants of K_1_ and K_2_ for carbonic acid dissociation [41].

### Measurements of photosynthetic oxygen evolution

The net photosynthetic rate of *U*. *linza* was measured by a Clark-type oxygen electrode (YSI Model 5300, USA). The thalli were prepared for about 1-cm-long segments with scissors, and the cutting damage was minimised by incubating the segments in cultured seawater for about 1 h. Approximately 0.015 g of harvested fresh weight algae was transferred to the oxygen electrode cuvette which containing 8 ml reaction medium, and the medium was stirred. The irradiance and temperature were maintained as the same as that in the growth condition. The measurement was finished within five minutes and the pH did not vary over this period. CA inhibitors (Sigma), acetazolamide (AZ) and 6-ethoxyzolamide (EZ) were used to study the mechanism of DIC acquisition via carbonic anhydrase (CA), and their final concentrations were both 200 μM. The buffer Tris, with a final concentration of 20 mM, was also used to test whether or not the extracellular acidic compartments present in the green macroalga (Fig 1).

To investigate the responses of *U*. *linza* grown under LC and HC to various pH and light levels, the net photosynthetic rates of thalli were also measured at different pH (7.0, 7.8, 8.2, 9.0 and 10.0) and light levels (LL, 100 μmol photons m^-2^ s^-1^; HL, 600 μmol photons m^-2^ s^-1^). The light level of 600 μmol photons m^-2^ s^-1^ was set as high light because it is the saturation light intensity for photosynthesis in *U*. *linza*, which was revealed in a preliminary experiment.

### Measurement of growth

The relative growth rate (RGR) of *U*. *linza* was estimated after the thalli had acclimated to the CO_2_ levels for three weeks as follows: RGR = (lnN_t_ − lnN_0_) / t × 100, where N_0_ is the initial fresh weight, N_t_ is the final fresh weight, and t is the number of culture days.

### Chlorophyll fluorescence measurements

Chlorophyll fluorescence parameters of *U*. *linza* grown under LC and HC were measured with a pulse modulation fluorometer (Water PAM, Walz). After dark adaption for 15 minutes, the induction curves of the thalli were carried out for determining the chlorophyll fluorescence parameters. The relative electron transport rate (rETR) and non-photosythetic quenching (NPQ) can be obtained from the induction curve. rETR, was calculated according to Genty *et al*. (1989). NPQ, was attained as fellows: NPQ = (F_m_ − F_m_’) / F_m_’, F_m_ and F_m_’ are the maximal fluorescence levels from algae after dark adaptation and in light respectively. The actinic light levels in the induction curves were set at 100 and 600 μmol photons m^-2^ s^-1^.

### Determination of photosynthetic pigments

Approximately 100 mg fresh weight of thalli was extracted with 10 mL of absolute methanol at 4°C for 24 h in darkness. The contents of Chl *a* and Chl *b* were estimated according to Wellburn [42].

### Statistical analysis

The data were presented as the means ± standard deviation (SD) of five replicates and analysed using the software SPSS v.21. The data under every treatment conformed to a normal distribution (Shapiro-Wilk, *P* > 0.05) and the variances could be considered equal (Levene’s test, *P* > 0.05). Independent samples t-tests were conducted to compare the seawater carbonate parameters, relative growth rate, Chl *a*, and Chl *b* between LC and HC. One-way ANOVAs were conducted to assess the differences in the net photosynthetic rate between inhibitors at LC and HC. Three-way ANOVA was conducted to investigate the effects of culture CO_2_, media pH, and light on net photosynthetic rate of *U*. *linza*. Two-way ANOVAs were conducted to assess the effect of culture CO_2_ and light on rETR and NPQ. Tukey HSD was conducted for *post hoc* investigation. The 95% confidence level was used in all analyses.

## Results

### Changes of seawater carbonate chemistry caused by ocean acidification

The effects of ocean acidification on seawater carbonate parameters were observed (Table 1). Projected ocean acidification decreased pH by 0.34 unit (Independent samples t-test, t = 29.242, df = 8, *P* < 0.001), CO_3_^2-^ by 50.43% (Independent samples t-test, t = 25.308, df = 8, *P* < 0.001) but increased *p*CO_2_ by 143.87% (Independent samples t-test, t = -22.777, df = 8, *P* < 0.001), DIC by 7.47% (Independent samples t-test, t = -6.023, df = 8, *P* < 0.001), HCO_3_^-^ by 9.94% (Independent samples t-test, t = -7.771, df = 8, *P* < 0.001), CO_2_ by 143.87% (Independent samples t-test, t = -22.779, df = 8, *P* < 0.001), with an insignificant effect on TA (Independent samples t-test, t = -1.369, df = 8, *P* = 0.208).

**Table 1:** Parameters of the seawater carbonate system at LC and HC. Measurements and estimation of the parameters are described in Materials and Methods. Data are the means ± SD (n = 5). LC, the low *p*CO_2_ condition, HC, the high *p*CO_2_ condition, DIC = dissolved inorganic carbon, TA = total alkalinity. The unit of *p*CO_2_ is μatm and all other parameters’ unit is μmol kg^-1^.Different superscript letters indicate significant differences between two conditions (*P* < 0.05).

Treatment	pH	*p*CO_2_	DIC	HCO_3_^-^	CO_3_^2-^	CO_2_	TA
LC	8.14±0.02^a^	404.9±20.6^a^	1982.1±32.8^a^	1846.5±31.8^a^	118.6±4.6^a^	17.0±0.9^a^	2152.6±33.4
HC	7.80±0.02^b^	987.4±53.4^b^	2130.2±44.1^b^	2030.0±42.2^b^	58.8±2.6^b^	41.4±2.3^b^	2186.2±43.6

### Effects of elevated CO_2_ on CCMs and growth

**Fig 2:**
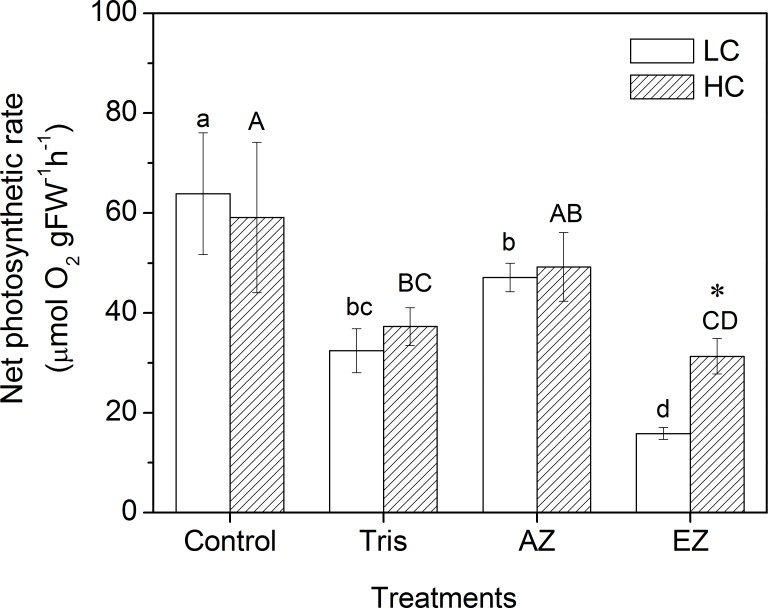
Net photosynthetic rate of *U*. *linza* grown at ambient (LC; 390 μatm) and elevated (HC; 1000 μatm) CO_2_ levels and measured under different treatments (Control, added with Tris, AZ, and EZ). Different superscript letters indicate significant differences between treatments within one culture condition (*P* < 0.05, one-way ANOVA and Tukey HSD test) and the lowercase and capital letters represent the comparisons at LC and HC, respectively. Asterisks indicate significant differences between culture conditions (*P* < 0.05, Independent samples t-tests).

**Fig 3:**
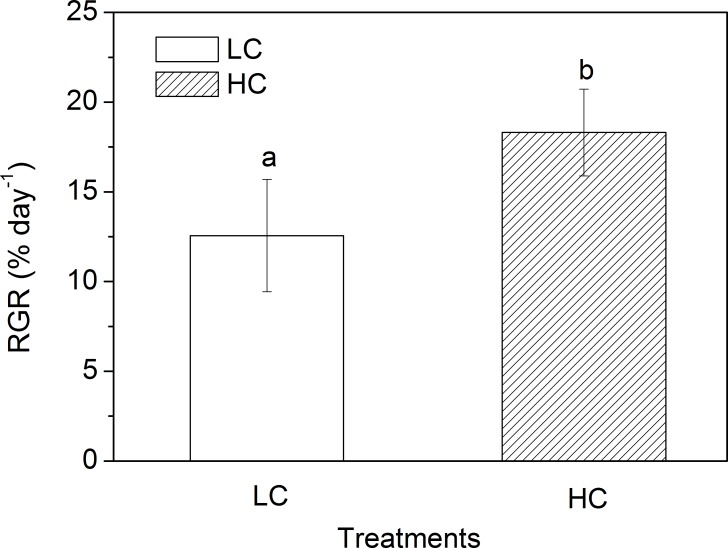
Relative growth rate of *U*. *linza* grown at ambient (LC; 390 μatm) and elevated (HC; 1000 μatm) CO_2_ levels. Different superscript letters indicate significant differences between culture conditions (*P* < 0.05, Independent samples t-tests).

The net photosynthetic rate of *U*. *linza* grown under LC was altered when inhibitors were added (Fig 2; ANOVA, *F* = 47.109, df = 3, 16, *P* < 0.001). It was reduced to 32.44 ± 4.40 μmol O_2_ g^-1^ FW h^-1^ from 63.87 ± 12.23 μmol O_2_ g^-1^ FW h^-1^ (Control) with the addition of Tris for thalli grown at LC (Tukey HSD, *P* < 0.001), which amounts to an inhibition rate of 49.21%. The net photosynthetic rate of *U*. *linza* grown at LC decreased to 47.10 ± 2.84 μmol O_2_ g^-1^ FW h^-1^ when AZ was added, indicating an inhibition rate of 26.26% (Tukey HSD, *P* = 0.005). Compared to Tris and AZ, EZ displayed a higher inhibition rate (75.19%) (Tukey HSD, *P* = 0.306), with the photosynthetic rate decreasing to 15.85 ± 1.20 μmol O_2_ g^-1^ FW h^-1^. The pattern varied for *U*. *linza* grown under HC. There was no significant difference in the net photosynthetic rate between the treatments of AZ (49.22 ± 6.87 μmol O_2_ g^-1^ FW h^-1^) and control (59.13 ± 15.04 μmol O_2_ g^-1^ FW h^-1^) (Tukey HSD, *P* > 0.05). Tris (Tukey HSD, *P* = 0.005) and EZ (Tukey HSD, *P* = 0.001) still had inhibitory effects on the net photosynthetic rate, with the inhibition rates of 37.00% and 47.03% respectively. But the difference in the net photosynthetic rate between Tris (37.25 ± 3.79 μmol O_2_ g^-1^ FW h^-1^) and EZ (31.32 ± 3.58 μmol O_2_ g^-1^ FW h^-1^) was insignificant (Tukey HSD, *P* = 0.705). HC did not increase the net photosynthetic rates of *U*. *linza* except under the addition of EZ (an increase of 97.64%, Independent samples t-test, t = -9.160, df = 8, *P* < 0.001). The effect of elevated CO_2_ on growth of *U*. *linza* was also examined (Fig 3). The RGR of thalli grown at LC was 12.56 ± 3.13% and the HC increased it to 18.31 ± 2.42% (Independent samples t-test, t = -3.250, df = 8, *P* = 0.012).

### Responses to seawater carbon chemistry and light

**Fig 4:**
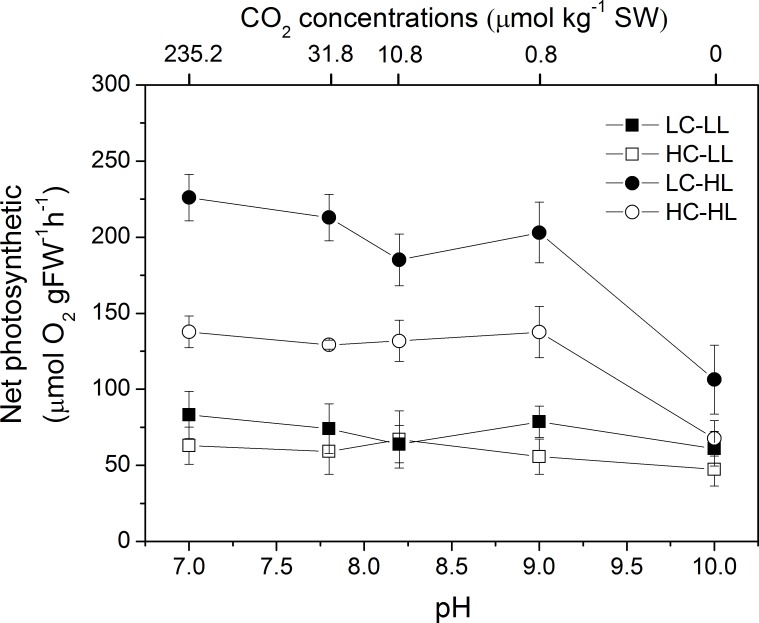
Changes of net photosynthetic rates of *U*. *linza* grown at ambient (LC; 390 μatm) and elevated (HC; 1000 μatm) CO_2_ levels measured at different pH and light (LL, 100 μmol photons m^-2^ s^-1^; HL, 600 μmol photons m^-2^ s^-1^) conditions.

The responses of *U*. *linza* grown at LC and HC to seawater carbon chemistry and light levels were investigated (Fig 4). There were no interactive effects of CO_2_, light, and seawater carbon chemistry but any two of them interacted on the net photosynthetic rate of *U*. *linza* (Table 2). For instance, HC did not affect the net photosynthetic rate of *U*. *linza* at LL but it significantly reduced net photosynthetic rate at HL, regardless of the seawater carbon chemistry conditions. Similarly, the differences in net photosynthetic rate between seawater carbon chemistry conditions at LL were insignificant but lower pH (or higher CO_2_) decreased net photosynthetic rate at HL.

**Table 2:** Three-way analysis of variance of the responses of the net photosynthetic rate in *U*. *linza* grown under LC and HC to various light and seawater pH levels. LC, the low *p*CO_2_ condition_,_ HC, the high *p*CO_2_ condition.

Source	Type III Sum of Squares	df	Mean Square	F	Sig.
CO_2_	34148.279	1	34148.279	83.052	<0.001
light	208338.621	1	208338.621	506.701	<0.001
pH	33933.106	4	8483.277	20.632	<0.001
CO_2_*light	13496.544	1	13496.544	32.825	<0.001
CO_2_*pH	4893.995	4	1223.499	2.976	0.024
light*pH	15493.055	4	3873.264	9.420	<0.001
CO_2_*light*pH	3143.366	4	785.842	1.911	0.117
Error	32893.313	80	411.166		

### rETR and NPQ

**Fig 5:**
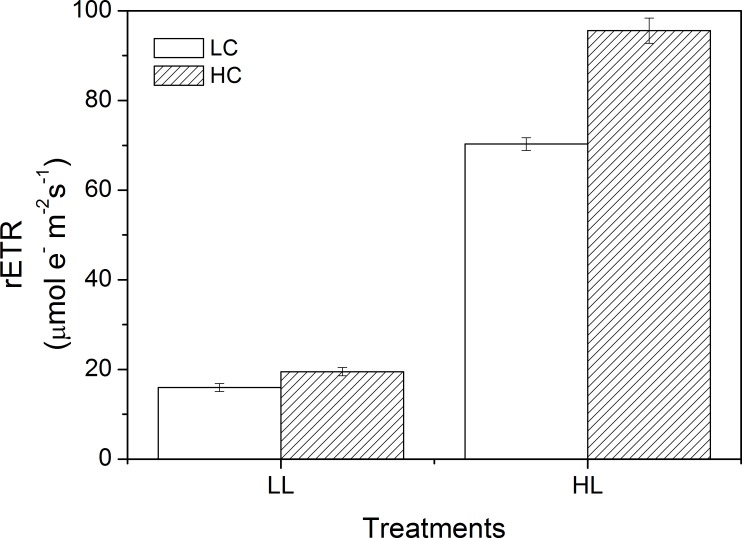
rETR of *U*. *linza* grown at ambient (LC; 390 μatm) and elevated (HC; 1000 μatm) CO_2_ levels and measured at different light conditions (LL, 100 μmol photons m^-2^ s^-1^; HL, 600 μmol photons m^-2^ s^-1^).

**Fig 6:**
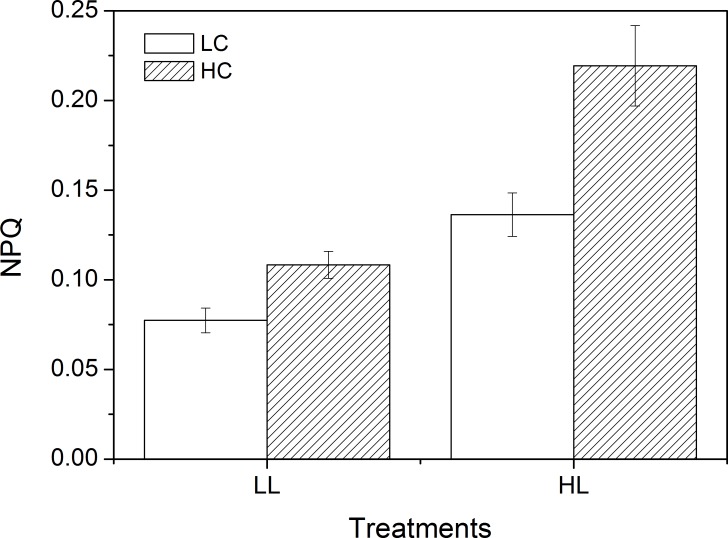
NPQ of *U*. *linza* grown at ambient (LC; 390 μatm) and elevated (HC; 1000 μatm) CO_2_ levels and measured at different light conditions (LL, 100 μmol photons m^-2^ s^-1^; HL, 600 μmol photons m^-2^ s^-1^).

There was an interactive effect on rETR between CO_2_ and light, and both also had main effects (Table 3). The rETR were 15.97 ± 0.90 μmol e^-^ m^-2^ s^-1^ (LC) and 19.50 ± 0.91 μmol e^-^ m^-2^ s^-1^ (HC) at LL and HL increased them to 70.28 ± 1.40 μmol e^-^ m^-2^ s^-1^ (LC) and 95.59 ± 2.82 μmol e^-^ m^-2^ s^-1^ (HC), respectively (Fig 5). HC increased rETR by 18.12% at LL and the stimulating effect (26.48%) was more significant at HL. CO_2_ and light also had an interactive effect on NPQ (Table 4). For instance, HC increased NPQ by 28.60% at LL while it was 37.82% at HL, indicating that the stimulating effect of CO_2_ increases with light (Fig 6). HL increased NPQ regardless of CO_2_.

**Table 3:** Two-way analysis of variance of the effects of CO_2_ and light on rETR in *U*. *linza*.

Source	Type III Sum of Squares	df	Mean Square	F	Sig.
CO_2_	1040.255	1	1040.255	359.624	<0.001
light	21256.621	1	21256.621	7348.574	<0.001
CO_2_*light	593.073	1	593.073	205.030	<0.001
Error	46.282	16	2.893		

**Table 4:** Two-way analysis of variance of the effects of CO_2_ and light on NPQ in *U*. *linza*.

Source	Type III Sum of Squares	df	Mean Square	F	Sig.
CO_2_	0.016	1	0.016	86.229	<0.001
light	0.036	1	0.036	191.977	<0.001
CO_2_*light	0.003	1	0.003	17.939	0.001
Error	0.003	16	<0.001		

#### Photosynthetic pigments

**Fig 7:**
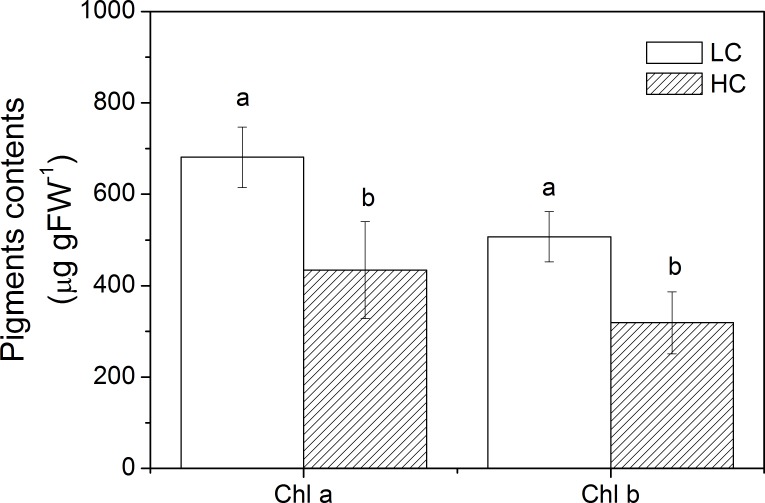
Contents of Chl *a* and Chl *b* in *U*. *linza* grown at ambient (LC; 390 μatm) and elevated (HC; 1000 μatm) CO_2_ levels. Different superscript letters indicate significant differences between culture conditions (*P* < 0.05, Independent samples t-tests).

The contents of Chl *a* and Chl *b* in *U*. *linza* grown at LC were 680.91 ± 65.81 μg g^-1^ FW and 507.06 ± 55.21 μg g^-1^ FW respectively and they decreased to 434.05 ±105.93 μg g^-1^ FW (Independent samples t-test, t = 4.426, df = 8, *P* = 0.002) and 318.67 ± 67.87 μg g^-1^ FW (Independent samples t-test, t = 4.815, df = 8, *P* = 0.001) in *U*. *linza* grown at HC (Fig 7).

## Discussion

### Ocean acidification changes CCMs

The inhibiting effect of Tris buffer on net photosynthetic rate of *U*. *linza* grown at LC implies that the thalli possess the acidic compartments as one of CCMs pathways. This is the first report of acidic compartments existing in cell wall of *Ulva* species, although it has been found in other macrophytes, such as *Ruppia cirrhosa* [43], *Zostera marina* [44], *Z*. *noltii* [45] and *Laminaria saccharina* [46]. Acidic compartments are created by excreting protons outside of the plasma membrane and thus CO_2_ concentration in the zones is enhanced above its level in the medium, which can speed up the diffusion of CO_2_ into the cell either through the cell membrane proper or via proteinaceous pores [46,47]. The Tris buffer is considered to act as a proton dissipating system facilitating the H^+^ diffusion out of the acidic compartments [46,48], which consequently increases the pH within acid compartments and weakens the function of acidic compartments as the CCMs pathway. The addition of AZ and EZ also reduced net photosynthetic rate of *U*. *linza* grown at LC, suggesting both extracellular CA and intercellular CA function in the CCMs of *U*. *linza*. Tris, AZ, and EZ inhibited the net photosynthetic rate of thalli grown at LC, which means that acidic compartments, external and internal CA work together in the thalli grown in natural seawater medium and the acquisition of exogenous carbon in the thalli may be achieved by the collaboration of acidic compartments and external CA.

After *U*. *linza* was cultured at HC for three weeks, the addition of Tris still inhibited net photosynthetic rate although the inhibition rate decreased from 49.21% at LC to 37.00%, indicating a little down-regulation of acidic compartments. In contrast, no significant difference in net photosynthetic rate of *U*. *linza* grown under HC was found when AZ was added, which suggests that the function of extracelluar CA should be completely switched off at HC. Mercado et al. [46] demonstrated that acidic compartments must feature external CA activity in *Laminaria saccharina* since the acidic compartments *per se* played a minor role when external CA was completely inhibited, but our study suggests that external CA is not essential for the acidic compartments in *U*. *linza*. When EZ is added, the net photosynthetic rate in *U*. *linza* at HC was reduced by 47.03%, lower than the decrease of 75.19% at LC, indicating intercellular CA was also down-regulated at HC. It is worth noticing that the net photosynthetic rate at HC was 97.64% higher than that at LC when EZ was added. This indicates that diffusion of CO_2_ plays a more important role at HC since the acquisition of exogenous carbon is mostly based on the diffusion of CO_2_ in seawater medium when activity of CA is inhibited. The acidic compartment at HC should contribute to the higher net photosynthetic rate under the treatment of EZ, and higher rETR at HC may supply additional ATP to maintain the operation of acidic compartment, considering energy is needed to run the proton pump in the acidic compartment. Although ocean acidification down-regulated CCMs and altered pathways of CCMs in *U*. *linza*, it did not increase or decrease the net photosynthetic rate. Neutral effects of elevated CO_2_ on photosynthesis were also found in phytoplankton assemblages [49] and macroalgae *U*. *prolifera* [38]. One possible reason is that photorespiration is also reduced along with the down-regulated CCMs at elevated CO_2_ conditions [50,51].

### Ocean acidification increases growth

The growth at HC was enhanced compared to LC (Fig 2), albeit the net photosynthetic rate in *U*. *linza* grown at HC did not increase. The enhancement of *Ulva* growth at HC might be attributed to the saved energy due to down-regulated CCMs, e.g., reduced synthesis of CA, which can benefit other metabolisms and biosynthesis. For example, high CO_2_ increased activity of nitrate reductase in *U*. *rigida* [52,53], brown seaweed *Hizikia fusiforme* [10], and calcifying rhodophyte *Corallina officinalis* [54]. Accordingly, the nitrogen assimilation in plants was enhanced at high CO_2_ conditions [10,52,53]. In addition to the saved energy from down-regulated CCMs, content of pigments (Chl *a* and Chl *b*) at HC was also decreased. Energy saved from the ‘pigment economy’ can also flow to other metabolisms and biosynthesis and hence enhance growth. The increased growth at HC was also reported in another green tidal alga, *U*. *prolifera* [38]. The similar physiological responses of the two main green tidal species to high CO_2_ level indicate that future ocean acidification might lead to more severe green tides when the light level is low.

### Responses of ocean acidification acclimatised thalli to seawater carbon chemistry and light

Under LL, the net photosynthetic rate of *U*. *linza* grown at LC was not affected by pH and even at the pH 10 where [CO_2_] was zero and [HCO_3_^-^] was below 10% (Fig 5), the net photosynthetic rate was still comparable to the values at other pH conditions. We presume that the robust CCMs at various pH conditions must be involved in two or more pathways for the acquisition of exogenous carbon. Our findings show that Tris, AZ and EZ all significantly decreased the net photosynthetic rate of *U*. *linza* (Fig 4), that is, acidic compartments, internal and external CA were all the functional elements in the CCMs. The robust CCMs of *U*. *linza* may partially explain its ecological success as it does experience pH perturbations in the field, which is lower pH at night due to respiration and higher pH in the daytime due to photosynthesis. When green tides break out, the seawater is covered with extensive *Ulva* thalli which could lead to a dramatic increase of pH, efficient CCMs at higher pH support continuous growth of *Ulva* and thus the development of green tides. High CO_2_ acclimatised *U*. *linza* still showed consistent net photosynthetic rate at various pH. As discussed above, ocean acidification altered the pathways of CCMs in *U*. *linza*, but it seems not to alter the capability of *U*. *linza* to respond to changes of seawater carbon chemistry, which could be attributed to its diverse pathways of CCMs and differential responses of pathways of CCMs to ocean acidulation.

On the other hand, the net photosynthetic rate in high CO_2_-cultured *U*. *linza* was reduced compared to the low CO_2_-cultured plant when they were exposed to high light intensity. When rETR and NPQ were examined, both of them were enhanced at HC and this trend was more significant at HCHL. NPQ is an important photoprotective process that can dissipate excess energy, and avoid or reduce formation of reactive oxygen species produced by excessive light. *U*. *linza* grown under HC showed increased NPQ (28.60%) when exposed to LL, suggesting that the down-regulated CCMs at HC may lead to the need to dissipate excess energy even at a light intensity of 100 μmol photons m^-2^ s^-1^. When thalli were exposed to 600 μmol photons m^-2^ s^-1^, HC-grown *U*. *linza* presented an increase of 37.82% in NPQ, indicating more severe light stress. Despite increased thermal dissipation (NPQ), high light still reduced the net photosynthetic rate of *U*. *linza* grown at HC, which may imply the protective activity is not enough to fully avoid the photodamage caused by excessive light. The increased NPQ and reduced net photosynthetic rate at HCHL was also documented in phytoplankton assemblages [37] and *U*. *prolifera* [38]. This assumption of thalli grown at HC suffering more severe light stress is also supported by the alteration of light-capture pigments. High CO_2_-grown *Ulva* had lower Chl *a* and Chl *b* contents. This decline in photosynthetic pigments could avoid over-excitation of the electron transport and be a sign of light sufficient in thalli grown at HC. Such a “pigment economy” phenomenon at elevated CO_2_ conditions seems to be a general rule in plants since it has been also found in *U*. *prolifera* [38], *U*. *rigida* [53], unicellular chlorophyte *Dunaliella viridis* [55], cyanobacterium *Spirulina platensis* [56], and trees [57]. In addition, highest pH of 10 dramatically reduced net photosynthetic rate when thalli were exposed to HL no matter *U*. *linza* was grown under LC or HC, indicating *U*. *linza* can maintain consistent net photosynthetic rate at various seawater carbon chemistry conditions when expose to LL but when light was enhanced, the increased requirement for CO_2_ was beyond the capacity of CCMs.

## Conclusions

Our study, for the first time, provided experimental data regarding the robustness of the ocean acidification acclimatised green tide algae to changes of seawater carbon chemistry and vulnerability to light stress and proposed a possible mechanism. Ocean acidification changed the CCMs of *U*. *linza*, in which the function of external CA was switched off, the proportion of internal CA was reduced, but the importance of acidic compartments and diffusion of CO_2_ was advanced, which may explain why ocean acidification did not affect the efficient response of the plant to various seawater carbon chemistry. The saved energy from the down-regulated CCMs may lead to increased vulnerability of the plant to high light. Future ocean surfaces might receive rising solar radiation with the compulsory implementation of air quality measures in industrialised countries and thus the decline in anthropogenic aerosol emissions [58]. From this point of view, future ocean acidification might have a depressing effect on *Ulva* and consequently *Ulva* cultivation or green tides.

## Supporting Information

S1 Dataset The whole dataset of this paper. (XLSX)
